# Notes and Comments

**Published:** 1893-07

**Authors:** 


### Notes and Comments.[1]

EDUCATION FOR ITS OWN SAKE.—In a recent editorial in *Harper's Magazine* the beauty of education and culture for its own sake is admirably expressed. The writer says, "If people could get the idea that what is called education is a good thing in itself, without reference to its practical uses, what a long step ahead the world would take! The notion that education must be for some definite purpose is responsible for much misdirected effort and many disappointments. What needs leavening and liberalizing and lifting up intellectually is the great mass of society. We shall get on a solid basis when we recognize the truth that a thorough education, a full development of all the faculties, is worth all it costs to the individual and to his or her associates, if it may never be put to any professional use." The infusion of culture into ordinary affairs in many towns and cities in the West is also alluded to. There is no doubt but this progressive spirit, with its many schemes for diffusing cultivation, is due to the fact that so many of our educated, well-bred young men and women have gone there and established themselves in business. To improve the tone of society, then, is excuse enough for the higher education, as the most important worldly thing is, how to make our lives and society most interesting and helpful.

THE THERAPEUTIC USE OF SYRUP OF CHLORIDE OF IRON (Ved.).—Owing to its destructive action on the enamel of the teeth and liability to disturbance of the stomach, the tincture of iron is objectionable. It may be advantageously replaced by the syrup. In this preparation the excess of acid is neutralized by an alkali, and while still presenting an acid reaction, it does not attack the teeth nor discolor the tongue. When it reaches the stomach it meets the free hydrochloric acid, and becomes therapeutically identical with the tincture. The syrup is more assimilable, and gives rise neither to nausea nor digestive trouble. Dose, one-half ounce, three times a day.—*Times and Register, from La Médecine Moderne.*

---

[1] The assistant editor solicits contributions for this department,—new methods, new remedies and formulas, or any short practical note which may prove of value to the practitioner or student. Address 1506 Arch Street, Philadelphia.

